# Pregnancy complications and loss: an observational survey comparing anesthesiologists and obstetrician–gynecologists

**DOI:** 10.1080/14767058.2024.2311072

**Published:** 2024-02-07

**Authors:** Natalie R. Barnett, Renuka M. George, Katherine H. Hatter, Norah R. Janosy, Samantha J. Vizzini, Shubhangi Singh, Rebecca E. Lee, Bethany J. Wolf, Camila Cabrera, Amy L. Duhachek-Stapelman, Daniel Katz

**Affiliations:** a Department of Anesthesia and Perioperative Medicine, Medical University of South Carolina, Charleston, SC, USA; b Department of Anesthesiology, University of Colorado, Children’s Hospital Colorado, Aurora, CO, USA; c Department of Anesthesiology, University of Virginia, Charlottesville, VA, USA; d Department of Anesthesiology, University of Michigan, Ann Arbor, MI, USA; e Department of Anesthesiology, Perioperative and Pain Medicine, Icahn School of Medicine at Mount Sinai, New York, NY, USA; f Department of Public Health Sciences, Medical University of South Carolina, Charleston, SC, USA; g Department of Obstetrics and Gynecology, Icahn School of Medicine at Mount Sinai, New York, NY, USA; h Department of Anesthesiology, University of Nebraska Medical Center, Omaha, NE, USA

**Keywords:** Abortion, spontaneous, occupational exposure, pregnancy complications, pregnancy outcome

## Abstract

**Objective:**

While there is increasing information regarding the occupational risks to pregnant physicians, there is inconsistent and limited subspecialty data. Physicians may be at increased risk for pregnancy complications due to occupational exposure, long work hours, nightshifts, and physical/mental demands. Additionally, little is known regarding the education physicians receive pertaining to pregnancy risks respective to their specialties as well as departmental/institutional support for pregnancy loss or complication. Therefore, a survey was developed and distributed across multiple academic sites to ascertain if there is an inherent occupation-associated risk of pregnancy complication(s) and/or pregnancy loss for anesthesiologists (ANES) when compared to obstetrician/gynecologists (OB/GYN).

**Methods:**

A specialty-specific survey was distributed electronically to attending ANES and OB/GYN, via departmental listservs at six participating academic medical centers. Responses were collected from March to October 2022 and included demographic information, practice characteristics, education about pregnancy risks and details of pregnancy complications and loss. The primary comparison between specialty groups was the occurrence of at least one pregnancy complication and/or loss. Logistic regression was used to evaluate specialty outcome associations. Additionally, complication rates and types between specialties were compared using univariate and multivariable models.

**Results:**

The survey was distributed to 556 anesthesiology and 662 obstetrics–gynecology faculty members with 224 ANES and 168 OB/GYN respondents, yielding an overall 32.2% response rate. Of the survey respondents, 103 ANES and 116 OB/GYN reported at least one pregnancy. Demographics were similar between the two cohorts. ANES had higher gravidity and parity relative to OB/GYN and tended to be earlier in their career at first pregnancy (*p* = .008, <.001, and .043, respectively). The rate of any pregnancy complication, including loss, was similar between specialties (65.1% (67/103) vs. 65.5% (76/116), *p* = .942). Of the respondents reporting at least one pregnancy, 56.7% of ANES and 53.9% of OB/GYN experienced a complication while at work. Obstetrician–gynecologists had higher use of reproductive assistance (28% (47/116) vs. 11% (20/103), *p* < .001). There were no notable differences between cohorts for complications, prematurity, and neonatal intensive care admission. Forty-one percent (161/392) of total respondents recalled learning about occupational risks to pregnancy, and ANES were more likely than OB/GYN to have recalled learning about these risks (121/224 (54%) and 40/168 (23.8%), respectively, *p* < .001).

**Conclusions:**

ANES and OB/GYN had similar risks for pregnancy complications and loss. Anesthesiologists were more likely to recall receiving education regarding occupational risk to pregnancy, though fewer than half of all survey respondents recalled learning about these risks. Our survey results are similar to the previously identified higher rate of pregnancy complications and loss in female physicians while uncovering areas of potential knowledge gaps for which institutions and practices could strive to improve upon. More research is needed to examine the relationship between occupation and pregnancy risk pertaining to female physicians with the goal being to identify modifiable risk factors.

## Introduction

Across the United States and worldwide, pregnancy and fertility are widely discussed in all realms, transcending cultural and socioeconomic groups. Within current medical literature, the risk workers exposed to the operating room (OR) environment is relatively unknown. Over 50 years ago, an initial study compared the pregnancy outcomes of registered nurses and physicians working in the OR to those outside the OR; both physicians and nurses in the OR exposure group experienced a statistically significant higher rate of miscarriage [1]. Interestingly, the higher rate of miscarriage in anesthesiologists as compared to the OR nurses was attributed to proximity to the anesthesia machine and exposure to waste anesthesia gases (WAGs) [1]. In fact, the National Institute for Occupational Safety and Health issued recommendations for exposure limits of nitrous oxide and halogenated anesthetics in 1977 in attempt to minimize WAG-associated risks, such as spontaneous abortion, stillbirth, and birth malformations [1].

With implementation of medical scavenging systems, the presumed occupational risk of WAG exposure has lessened, but the health and pregnancy risks associated with the perioperative environment remain relatively unknown. A recent study found an almost twofold increase in pregnancy complications in surgeons compared to other female physicians [2]. This may be influenced additionally by long work hours, a challenging work environment, other occupational hazards, and/or chemical and infectious exposures, specifically antineoplastic agents, disinfectants, and WAG [3–8]. This multi-center self-report survey study was designed in attempt to further elucidate any differences in pregnancy complication and loss experienced by anesthesiologists (ANES) and obstetricians/gynecologists (OB/GYN). Both physician groups have similar occupational physical and mental demands including long work hours, prolonged standing and lifting, unpredictable and variable schedules including overnight shifts. Additionally, both groups have significant OR exposure with the associated environmental risks, with ANES typically in closer proximity to the anesthesia machine. It is critical to understand if, like their surgical colleagues, they have similar or different risks to pregnancy.

## Methods

The study was approved by the appropriate Institutional Review Board (IRB) at each participating site and the requirement for written informed consent was waived by the IRB at each individual site. This manuscript adheres to the applicable STROBE guidelines.

The primary goals of the study were to (1) estimate the prevalence of pregnancy complications in ANES and (2) to compare that prevalence to OB/GYN. OB/GYN was chosen as a comparative group given the relatively increased proportion of female physicians within the specialty and overall large specialty size, increasing the potential sample size. Most surgical specialties are vastly subspecialized and would potentially add many more confounders. To obtain data, an email was distributed to the ANES and OB/GYN faculty listservs at six participating academic medical centers: the Medical University of South Carolina, the Icahn School of Medicine at Mount Sinai, University of Colorado, University of Virginia, University of Nebraska Medical Center, and University of Michigan from March to October 2022. These sites were chosen based on geographic location and professional contacts. The survey was distributed to attending physicians only and was sent on three separate occasions to each participating institution. Prior to release, the survey was informally piloted throughout the survey design process by the authors and faculty members from participating sites using a modified Delphi approach until agreement was found on all items. Six authors participated in the modified Delphi process over a six-month period to identify primary topics of investigation, organization of survey, and total included questions. The survey examined the formality of educational setting, the type of educator, and the quality of the information (surveys available as Supplemental Surveys 1 and 2).

### Power calculation and statistical methods

Based upon the number of faculty and staff in the ANES and OB/GYN departments at the included institutions, an *a priori* sample size calculation found a sample size of 130 ANES would allow us to estimate a two-sided 95% confidence interval pregnancy complication rate with a width no greater than 0.18. For comparison of the rates of complication between specialties, a sample of 145 OB/GYN and 130 ANES would provide 80% power to detect a difference in pregnancy complication rates of ≥16.6% using a two-sided test and significance level *α* = 0.05.

Descriptive statistics were calculated for all participant demographics and practice characteristics. Primary outcomes of interest include knowledge of potential risks to pregnancy related to practitioner specialty and occurrence of at least one pregnancy complication by specialty. The association between these outcomes by specialty was examined using a logistic regression approach. We also examined univariate associations between additional respondent characteristics with each outcome and developed multivariable models using backwards selection retaining specialty (as our main variable of interest) and all additional variables significant at *p* < .05. All analyses were conducted in SAS v. 9.4 (SAS Institute, Cary, NC).

## Results

**Figure 1.**
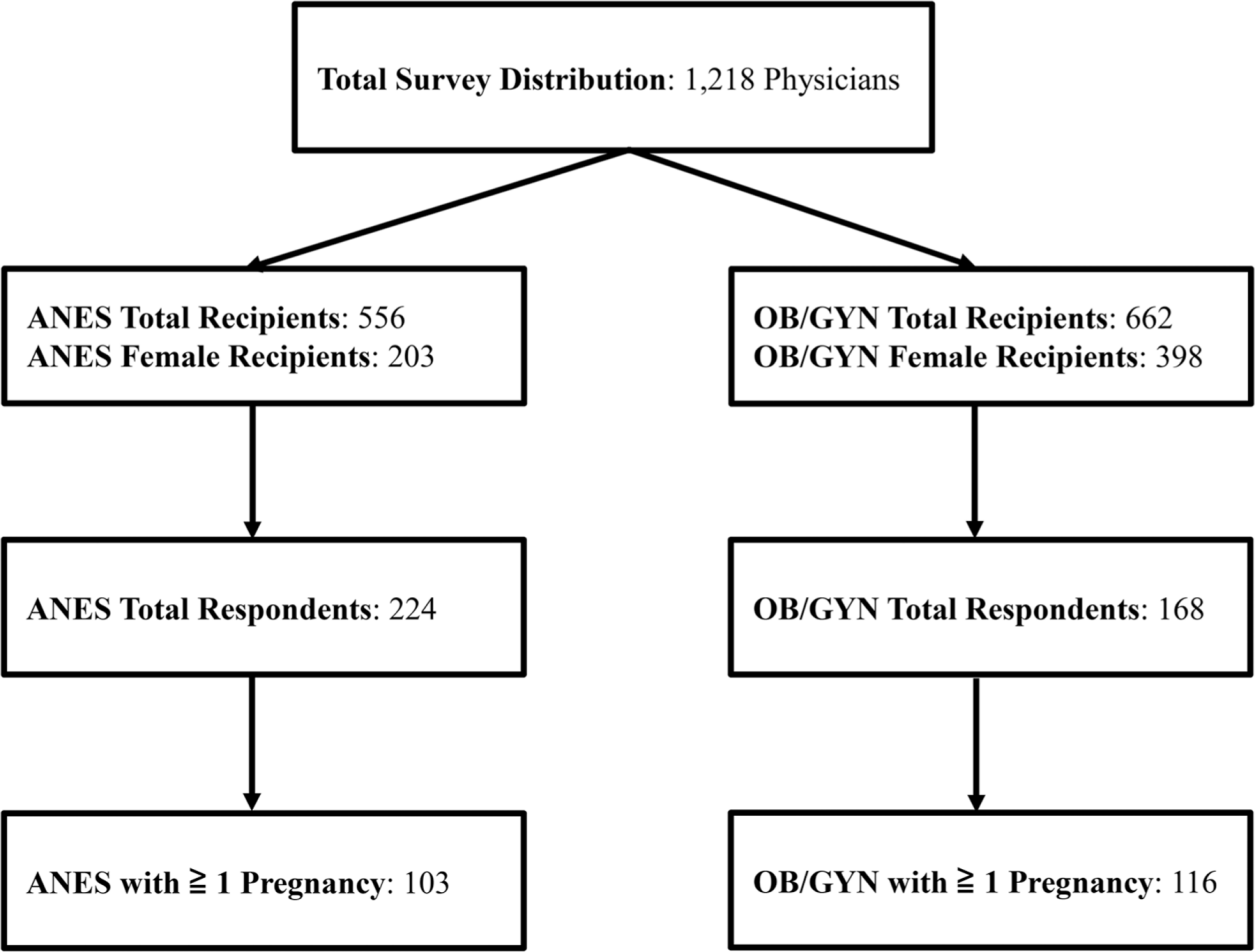
Consort diagram.

The survey was distributed to 556 ANES attending physicians of which 203 recipients were female, and 662 OB/GYN attending physicians of which 398 recipients were female. The final study population reflected a 32.2% (392/1218) response rate and included 224 ANES (224/556, or 40.3%) of whom 103 reported at least one pregnancy and 168 OB/GYN (168/662, or 25.4%) of whom 116 reported at least one pregnancy (Figure 1) and were included in analysis for pregnancy complication and/or loss. Participant demographic and practice characteristics by provider type are provided in Table 1. The age and self-reported race of ANES and OB/GYN providers at time of survey completion were similar.

### Complications risks among those with at least one pregnancy

Of the 224 ANES respondents and the 168 OB/GYN respondents, 45.98% (103/224) and 69.05% (116/168) respectively reported experiencing at least one pregnancy. Characteristics of respondents who reported one or more pregnancy by provider type are shown in Table 2. ANES had higher gravidity and parity relative to OB/GYN and tended to be earlier in their career at first pregnancy (*p* = .008, <.001, and .043, respectively). Boxplots of the distribution of gravidity and parity by respondent group are demonstrated in Supplemental Figure 1. Of the respondents who reported a pregnancy, OB/GYN were more likely than ANES to have utilized reproductive assistance, 28% (47/168) versus 11.6% (26/224), respectively (*p* < .001). There was no difference between groups regarding type of reproductive assistance.

There was no significant difference between groups in the reporting of any complications and/or losses, or number of complications and/or losses. Sixty-five percent of both the ANES and OB/GYN groups reported one or more pregnancy complications or losses with a median number of 1 [2;0–6]. There was not a significant difference found in occurrence of specific types of complications (i.e. subchorionic hematoma, placental abruption, preeclampsia, preterm labor, or preterm membrane rupture). A greater proportion of ANES reported experiencing one or more pregnancy losses, though the difference was not statistically significant (*p* = .057, 35.9% versus 24.1%). Rates of reported congenital abnormalities, genetic syndromes, premature infants, and infants requiring admission to the neonatal intensive care unit were similar between ANES and OB/GYN.

Univariate associations between participant characteristics with report of one or more pregnancy complications or loss were also examined. Higher gravidity was the only variable associated with increased odds of reporting any complication or loss (*p* < .001, odds ratio (95% CI): 1.80 (1.37, 2.35)). In the univariate model, there was no significant difference in the odds of reporting any complication or loss between ANES and OB/GYN (*p* = .942; OR (95% CI): 0.98 (0.56, 1.71)). The multivariable model of complication occurrence included specialty (as the main variable of interest) and gravidity after stepwise removal of variables. Specialty remained non-significant after controlling for gravidity (*p* = .363). In individuals with at least one reported pregnancy, increase in gravidity had more than 85% higher odds of experiencing a complication after controlling for specialty. Odds ratios and 95% confidence intervals for the univariate and multivariable models of pregnancy complication occurrence are shown in Supplemental Table 1. Age at first pregnancy was not associated with complication rates for the whole cohort or by specialty (Supplemental Table 2).

### Experiences if complication or loss happened at work

Sixty-seven of the 103 ANES respondents and 76 of the 116 OB/GYN respondents who had one or more pregnancies experienced a complication or loss. Among those who reported a complication or loss, 56.7% (38/67) of ANES and 53.9% (41/76) of OB/GYN experienced it while at work. Therefore, of the total respondents who reported having had one or more pregnancies, 36% experienced a loss or pregnancy complication while at work. Of the 79 respondents (both ANES and OB/GYN) who experienced a loss or complication while at work, 72% (57/79) informed their supervisor of the complication. Nine of the respondents who experienced the complication or loss at work were unable to leave to seek medical care. Among those who reported experiencing a complication or loss while at work, there were no differences between ANES and OB/GYN in the proportion that reported the issue to their employer or in the ability for the physician to leave work and receive medical care (Table 3). A significantly greater proportion of ANES respondents reported that their supervisor was male at the time of the reported complication or loss as compared to OB/GYN (*p* < .001).

### Knowledge regarding pregnancy risks related to specialty

Among the 392 total ANES and OB/GYN respondents, 41% (161/392) recalled having learned about potential risks to pregnancy related to their specialty. ANES were more likely to have learned about these risks than OB/GYN (121/224 ANES respondents (54%) versus 40/168 OB/GYN respondents (23.8%), respectively, *p* < .001). However, less ANES respondents reported learning through a grand rounds environment than OB/GYN (30/121 (24.8%) versus 25/40 (62.5%), respectively, *p* < .001). Of the 161 total respondents who reported having learned of potential occupational risks to pregnancy, 34% (55/161) reported learning through grand rounds, 32% (52/161) reported learning through institutional training, 56% (90/161) reported learning through informal conversations, 52% (84/161) reported learning through self-research, and 17% (28/161) did not recall where they had learned about potential risks. Univariate and multivariate models were created to investigate potential relationships between types of education regarding occupational pregnancy risk and demographics between the two cohorts and can be found in Supplemental Table 3.

## Discussion

Routine occupational activities for ANES and OB/GYN physicians such as heavy lifting, prolonged standing/walking, and prolonged bending have been associated with pregnancy complications including preterm delivery, miscarriage, preeclampsia, low birth weight, and small for gestational age [4,8]. Our data did not identify statistically significant differences in pregnancy risk between cohorts, though ANES respondents experienced first pregnancy earlier in their career despite being of similar ages. We also found that OB/GYN reported a higher use of reproductive assistance, perhaps secondary to both knowledge and access. While previous studies have reported an overall higher rate of pregnancy complications in physicians [9], our findings in surveyed ANES (65.1%) and OB/GYN (65.5%) are higher than those survey results reported in surgeons (48.3%) and surgical specialties, such as orthopedics (31%) and urology (25%) [2,7,10–12].

Of the total survey respondents, 65% of those with at least one known pregnancy reported experiencing a pregnancy complication or loss, which appears to be higher than the 11–23% that has been reported in the general population (Supplemental Table 4) [13–19]. When specifically assessing pregnancy loss, 36% of ANES and 24% of OB/GYN respondents reported at least one known pregnancy loss, whereas the overall reported incidence of pregnancy loss ranges from 8 to 15% of clinically recognized pregnancies [13,16–19]. These observations could potentially be skewed due to the different types of reporting between our survey study and the current literature. Survey associated recall, response bias, and overall survey response rate could also affect potential comparisons.

Over half of respondents who experienced a complication or loss, experienced it while at work (57% ANES and 54% OB/GYN). Of those who experienced a loss or complication while at work, 28% did not alert their supervisor. Moreover, 80% of ANES and 91% of OB/GYN did not leave work to seek medical care. Respondents were not specifically questioned whether a request was made but denied. However, 100% of physicians experiencing a significant health event should be granted the ability to seek urgent or emergent medical care, regardless of the type of event. Furthermore, only about a third of both groups requested a leave of absence for the complication or loss. The potential physical sequelae of miscarriage are well-known, such as bleeding and infection, whereas the psychological consequences are less well understood and can be substantial [13,20,21]. In fact, there is a strong association of anxiety, post-traumatic stress disorder, depression, and even suicide following pregnancy loss [13,20,21]. Therefore, it is not unreasonable to request a leave of absence for such an event and subsequent follow-up medical care.

Despite available occupational risk literature, only 41% of respondents recalled learning about potential specialty-related risks to pregnancy. Given historic WAG interest, it is not surprising that ANES had 4×-increased odds in learning about risks. Most respondents reported informal learning through conversations or self-directed research, whereas far less received formal education. This is surprising, as most institutions have multiple educational avenues such as departmental grand rounds and hospital-wide electronic learning modules. Because we surveyed attending physicians only, we do not have data on how nurses, surgical technicians, residents, and students are educated. Given all the potential health care workers exposed to OR environment-related occupational health risks with physically demanding job responsibilities, there is distinct opportunity for multidisciplinary education.

A strength of this study includes the multi-center design. This allows for a broader cross-section of potential responses both geographically and practice type. This aids in minimizing center-specific culture surrounding pregnancy and pregnancy complication as well as educational policies. Additionally, the inter-disciplinary nature of the study highlights that educational lapses exist across both specialties. The survey was sent a total of three times to all institutions in order to maximize response rate.

There are certainly multiple study limitations. First, respondents were not questioned on health factors known to contribute to pregnancy complication and/or loss such as physical exercise habits, and tobacco, alcohol and/or illicit drug use. Second, there are inherent risks to a survey-based study including reliability, recall, and self-reporting bias. The anonymous nature of the survey should help mitigate self-report bias, but, for many, this experience is traumatic and may limit responses despite anonymity (non-response bias). Pregnancy during residency versus attending physician was not surveyed and analyzed due to the already expansive data points collected, though there may have been a potential influence of increased workload during residency. However, we did attempt to capture early versus late career by stratifying based upon age greater or less than thirty years at first pregnancy (Supplemental Table 2). We chose to only include attending physicians to minimize potential confounding differences between advanced practice nurses, residents, and medical students. Finally, our overall response rate was 32.2% yielding a small sample size and potentially limits the generalizability to the anesthesiology and obstetrician gynecology populations at large, as well as limiting power analysis in the instance of small absolute number of specific complications. Potential contributing factors for this response rate include post-COVID burnout and periods of short staffing limiting faculty engagement, non-response from faculty that are not invested in the topic (i.e. male faculty), large percentage of anesthesiologist authors as opposed to obstetricians which may limit willingness of response to an outside department (overall response was 40.3% versus 25.4%, respectively), and finally the fundamentally sensitive nature of the topic at hand.

Our survey results did not reveal any differences in pregnancy risk between ANES and OB/GYN. It should be noted, however, that occupational risk to pregnancy education appears to be significantly lacking. Additionally, it is unclear based on our survey results whether those women who experienced a pregnancy loss or complication while at work were able to leave work to seek medical care, should they have requested to do so. Given the known physical and mental health consequences of pregnancy loss, occupational support from the onset of complication and/or loss could be beneficial to these women. Finally, while our survey results did not reveal a difference in pregnancy loss or complication between specialties, it did suggest that there is an opportunity for improved education and leave policies for physicians experiencing these events.

## Supplementary Material

MFMSuppTable1

MFMSuppTable2

MFMSuppTable3

MFMSuppTable4

PregOutAnesthesiaProvider

PregOutOBprovider

V2SuppTable1

SuppFig1

## Figures and Tables

**Table 1.** Participant characteristics by provider type.

	Anesthesia (*N* = 224)	OB/GYN (*N* = 168)	*p*
Age at time of survey, *N* (%)			.517
25–30 years old	2 (0.89)	6 (3.57)	
31–35 years old	48 (21.4)	39 (23.2)	
36–40 years old	51 (22.8)	40 (23.8)	
41–45 years old	54 (24.1)	15 (8.93)	
46–50 years old	24 (10.7)	16 (9.52)	
51–55 years old	13 (5.80)	19 (11.3)	
56–60 years old	9 (4.02)	16 (9.52)	
>61 years old	23 (10.3)	17 (10.1)	
Race, *N* (%)			.861
White	177 (79.0)	130 (77.4)	
Black	5 (2.23)	6 (3.57)	
Asian	21 (9.38)	19 (11.3)	
Other	11 (4.91)	7 (4.17)	
Not answered	10 (4.46)	6 (3.57)	
Hispanic ethnicity, yes, *N* (%)	8 (3.57)	10 (5.95)	.230
Years in practice (including residency), *N* (%)^a^			.046
0–5 years	18 (8.07)	17 (10.1)	
6–10 years	72 (32.3)	45 (26.8)	
11–15 years	49 (22.0)	34 (20.2)	
16–20 years	40 (17.9)	12 (7.14)	
21–25 years	10 (4.48)	16 (9.52)	
>25 years	34 (15.2)	44 (26.2)	
How would you describe your practice? *N* (%)			<.001
Academic	218 (97.3)	131 (78.0)	
Private	2 (0.89)	32 (19.1)	
Per diem/other	3 (1.34)	5 (2.98)	
What percentage of your cases do you supervise residents/CRNAs/AAs/NPs/PAs? *N* (%)^a^			<.001
0–25%	12 (5.41)	34 (20.2)	
26–50%	27 (12.2)	16 (9.52)	
51–75%	30 (13.5)	22 (13.1)	
>75%	150 (67.6)	83 (49.4)	
Do not supervise	3 (1.35)	13 (7.74)	
What percentage of your attending colleagues are female? *N* (%)^a^			<.001
0–25%	14 (6.33)	5 (3.01)	
26–50%	161 (72.8)	4 (2.41)	
51–75%	45 (20.4)	80 (48.2)	
>75%	1 (0.45)	77 (46.4)	
Do you recall learning about potential risks to pregnancy related to your specialty? Yes, *N* (%)^a^	121 (54.3)	40 (24.0)	<.001
If Yes, where, *N* (%)			
Grand rounds	30 (24.8)	25 (62.5)	<.001
Institutional training	34 (28.1)	18 (45.0)	.074
Informal conversation	71 (58.7)	19 (47.5)	.169
Self-research	58 (47.9)	26 (65.0)	.091
Do not recall	27 (22.3)	1 (2.50)	.003
Are you aware of a policy at your institution for support for pregnant employees? Yes, *N* (%)	90 (40.2)	42 (25.0)	.007
Are you aware of a policy at your institution for education on specialty related risks for pregnant employees? Yes, *N* (%)	20 (8.93)	7 (4.17)	.180
Are you currently using or have you ever used reproductive assistance? Yes, *N* (%)	26 (11.61)	47 (27.98)	<.001
Type of assistance, *N* (%)			
Oral medication	9 (34.6)	19 (40.4)	.812
*In vitro* fertilization	13 (50.0)	24 (51.1)	1.000
IUI	12 (46.2)	19 (40.4)	.821
Surrogacy	0 (0.00)	1 (2.13)	1.000
Other	3 (11.5)	11 (23.4)	.352
Rounds oral medication, median (IQR; range)	2.5 (1.25; 1–7)	3.0 (3.75; 1–10)	.648
Rounds *in vitro*, median (IQR; range)	2 (3; 1–7)	2 (2; 1–9)	.408
Rounds IUI, median (IQR; range)	2 (1.25; 1–6)	2 (2; 1–12)	.913
Have you ever been pregnant, yes, *N* (%)	103 (45.98)	116 (69.05)	<.001

a Between 1 and 5 providers did not provide a response to this question.

**Table 2.** Characteristics of those reporting at least one pregnancy by provider type.

	Anesthesia (*N* = 103)	OBGYN (*N* = 116)	*p*
Gravidity, median (IQR; range)	3 (1.5; 1–7)	2 (2; 1–10)	.008
Parity, median (IQR; range)	2 (1; 0–4)	2 (1; 0–7)	<.001
Any complications or loss, yes, *N* (%)	67 (65.1)	76 (65.5)	.942
# Complications/losses, median (IQR; range)	1 (2; 0–6)	1 (2; 0–6)	.597
Age at first pregnancy, *N* (%)			.453
15–20	0 (0.00)	1 (0.86)	
21–25	6 (5.83)	3 (2.58)	
26–30	34 (33.0)	32 (27.6)	
31–35	52 (50.5)	62 (53.4)	
36–40	11 (10.7)	16 (13.8)	
40–45	0 (0.00)	2 (1.72)	
Training at time of pregnancy, *N* (%)			.043
Pre-residency	12 (11.7)	6 (5.17)	
Residency	42 (40.8)	34 (29.3)	
Fellowship	8 (7.77)	19 (16.4)	
Attending	40 (38.8)	55 (47.4)	
Other	1 (0.97)	2 (1.72)	
Type of complication, yes, *N* (%)			
Subchorionic hematoma	6 (5.83)	10 (8.62)	.451
Placental abruption	5 (4.85)	5 (4.31)	1.000
Preeclampsia	8 (7.77)	11 (9.48)	.811
Preterm labor	19 (18.4)	15 (12.9)	.270
Preterm membrane rupture	8 (7.77)	7 (6.03)	.790
Pregnancy loss ever	37 (35.9)	28 (24.1)	.057
Other complication	21 (20.4)	30 (25.9)	.423
# Losses, median (IQR; range)	0 (1; 0–5)	0 (0; 0–4)	.146
# Losses among those with at least 1 loss, median (IQR; range)	1 (1; 1–5)	2 (2; 1–4)	.145
Congenital abnormality? Yes, *N* (%)^a^	7 (6.93)	6 (5.31)	.620
Genetic syndrome? Yes, *N* (%)^a^	7 (6.80)	5 (4.50)	.559
Premature baby? Yes, *N* (%)^a^	22 (21.4)	23 (20.0)	.805
NICU stay? Yes, *N* (%)^a^	22 (21.6)	22 (19.3)	.679

a Between 1 and 5 providers did not provide a response to this question.

**Table 3.** Experience among those who experienced a complication at work.

	Anesthesia (*N* = 67)	OBGYN (*N* = 76)	*p*
Did you experience the complication while at work? Yes, *N* (%)	38 (56.7)	41 (53.9)	.87
If the complication happened at work:			
Did you alert your employer of the complication? Yes, *N* (%)	29 (76.3)	28 (68.3)	.427
Was your supervisor at the time male or female? Male, *N* (%)^a^	30 (85.7)	17 (41.5)	<.001
Were you able to leave and seek medical care? Yes, *N* (%)^b^	24 (80.0)	30 (90.9)	.289
Did you request a leave of absence for this complication? Yes, *N* (%)^c^	21 (33.3)	26 (36.1)	.735

a Three anesthesia providers who experienced a complication at work did not answer this question.

b Eight anesthesia providers and eight OBGYN providers who experienced a complication at work did not answer this question.

c Four anesthesia providers and four OBGYN providers who experienced a complication did not answer this question.

## Data Availability

The data that support the findings of this survey study are available upon request from the corresponding author, NRB. The data are not publicly available due to the sensitive nature of the study and concerns for the privacy of research participants.
