# Supplementary material for: Pregnancy complications and loss: an observational survey comparing anesthesiologists and obstetrician–gynecologists
Source: J Matern Fetal Neonatal Med. Author manuscript; Available in PMC 2025 Dec 1. (PMC11234813; doi:10.1080/14767058.2024.2311072)
Supplement: MFMSuppTable1 [file NIHMS2004406-supplement-MFMSuppTable1.docx]

**Supplemental Table 1**: Univariate and multivariable models of any pregnancy complication or loss among participants who report ever being pregnant. Values reported are odds ratios with 95% confidence intervals.

|  | **Univariate** | | **Multivariable** | |
| --- | --- | --- | --- | --- |
| **Variable** | **OR (95% CI)** | **P** | **OR (95% CI)** | **P** |
| Provider Type, Anesthesiologist vs. OB | 0.98 (0.56, 1.71) | 0.942 | 0.76 (0.41, 1.38) | 0.363 |
| Gravidity, increase of 1 | 1.80 (1.37, 2.35) | <0.001 | 1.85 (1.40, 2.44) | <0.001 |
| Parity, increase of 1 | 1.24 (0.92, 1.66) | 0.163 |  |  |
| Attending/Fellow vs. Resident/ Pre-resident | 0.74 (0.42, 1.30) | 0.296 |  |  |
