# Supplementary material for: Pregnancy complications and loss: an observational survey comparing anesthesiologists and obstetrician–gynecologists
Source: J Matern Fetal Neonatal Med. Author manuscript; Available in PMC 2025 Dec 1. (PMC11234813; doi:10.1080/14767058.2024.2311072)
Supplement: MFMSuppTable2 [file NIHMS2004406-supplement-MFMSuppTable2.docx]

**Supplemental Table 2**:

Complication rates by age at first pregnancy (< 30 years vs. > 30 years) for all providers and by provider type. Values are reported as N (%).

| **Age at First Pregnancy** | **All Providers**  **(N_<30_ =76; N_>30_ = 143)** | **Anesthesia**  **(N_<30_ =40; N_>30_ = 63)** | **OBGYN**  **(N_<30_ = 36; N_>30_ = 80)** |
| --- | --- | --- | --- |
| ≤ 30 year | 50 (65.8%) | 25 (62.5%) | 25 (69.4%) |
| > 30 year | 93 (65.0%) | 42 (66.7%) | 51 (63.8%) |
