# Supplementary material for: Pregnancy complications and loss: an observational survey comparing anesthesiologists and obstetrician–gynecologists
Source: J Matern Fetal Neonatal Med. Author manuscript; Available in PMC 2025 Dec 1. (PMC11234813; doi:10.1080/14767058.2024.2311072)
Supplement: MFMSuppTable3 [file NIHMS2004406-supplement-MFMSuppTable3.docx]

**Supplemental Table 3:** Univariate and Multivariable models of knowledge regarding pregnancy risk. Values reported are odds ratios with 95% confidence intervals.

|  | **Univariate** | | **Multivariable** | |
| --- | --- | --- | --- | --- |
| **Variable** | **OR (95% CI)** | **P** | **OR (95% CI)** | **P** |
| Provider Type, Anesthesiologist vs. OB | 3.77 (2.42, 5.86) | <0.001 | 4.08 (2.52, 6.62) | <0.001 |
| Years in practice, ~5 year increase | 1.26 (1.11, 1.43) | <0.001 | 1.39 (1.21, 1.60) | <0.001 |
| Ever Pregnant, Yes vs. No | 0.41 (0.27, 0.62) | <0.001 | 0.48 (0.31, 0.75) | 0.001 |
| >50% of practice is female vs. <50% | 0.53 (0.35, 0.88) | 0.002 |  |  |
| Academic practice vs. other | 2.22 (1.08, 4.54) | 0.030 |  |  |
