# Supplementary material for: Pregnancy complications and loss: an observational survey comparing anesthesiologists and obstetrician–gynecologists
Source: J Matern Fetal Neonatal Med. Author manuscript; Available in PMC 2025 Dec 1. (PMC11234813; doi:10.1080/14767058.2024.2311072)
Supplement: MFMSuppTable4 [file NIHMS2004406-supplement-MFMSuppTable4.docx]

**Supplemental Table 4**: Complication incidences of ANES, OB/GYN, and general population

|  | ANES  (N = 103) | OB/GYN  (N = 116) | General Population |
| --- | --- | --- | --- |
| Any complications n (%) | 67 (65.1) | 76 (65.5) | 11* -19.1** |
| Pre-eclampsia n (%) | 8 (7.77) | 11 (9.48) | 1.2^1^-4.6^2^ |
| Prematurity n (%) | 22 (21.4) | 23 (20.0) | 9.62^3^ |
| Subchorionic hemorrhage n (%) | 6 (5.83) | 10 (8.62) | 9^4^-12.5^5^ |
| Placental abruption n (%) | 5 (4.85) | 5 (4.31) | 0.4-1^6^ |
| Pregnancy loss n (%) | 37 (35.9) | 28 (24.1) | 10^7^ -13.5^8^ |
| NICU Admission n (%) | 22 (21.6) | 22 (19.3) | 9.07^9^ |
| Genetic Disorders n (%) | 7 (6.8) | 5 (4.50) | 0.0017-0.005†; 0.0014†† |
| Congenital abnormality n (%) | 7 (6.93) | 6 (5.31) | 2.03- 2.22^10^ |
| * Patient population from United States, complications included pre-eclampsia and eclampsia, isolated hypertension of pregnancy, postpartum hemorrhage, anesthesia-related complications, diabetes mellitus, venous thromboembolic disease, stroke, severe sepsis and septic shock, injuries, status asthmaticus, amniotic fluid embolism, acute myocardial infarction and ischemia, aortic aneurysm and dissection, obstetrical air embolism, status epilepticus, diabetic coma, and obstetrical pyemic and septic embolism^11^  ** Patient population from Sweden, complications included preeclampsia or eclampsia, gestational HTN, gestational diabetes, preterm birth, small for gestational age, and stillbirth^12^  † Autosomal recessive diseases †† Autosomal dominant diseases^13^ | | | |

1. Raio L, Bolla D, Baumann M. Hypertension in pregnancy. *Curr Opin Cardiol*. Jul 2015;30(4):411-5. doi:10.1097/HCO.0000000000000190

2. Ben-Haroush A, Yogev Y, Mashiach R, Meizner I. Pregnancy outcome of threatened abortion with subchorionic hematoma: possible benefit of bed-rest? *Isr Med Assoc J*. Jun 2003;5(6):422-4.

3. Mayrink J, Costa ML, Cecatti JG. Preeclampsia in 2018: Revisiting Concepts, Physiopathology, and Prediction. *ScientificWorldJournal*. 2018;2018:6268276. doi:10.1155/2018/6268276

4. Purisch SE, Gyamfi-Bannerman C. Epidemiology of preterm birth. *Semin Perinatol*. Nov 2017;41(7):387-391. doi:10.1053/j.semperi.2017.07.009

5. Inman ER, Miranian DC, Stevenson MJ, Kobernik EK, Moravek MB, Schon SB. Outcomes of subchorionic hematoma-affected pregnancies in the infertile population. *Int J Gynaecol Obstet*. Dec 2022;159(3):743-750. doi:10.1002/ijgo.14162

6. Tikkanen M. Placental abruption: epidemiology, risk factors and consequences. *Acta Obstet Gynecol Scand*. Feb 2011;90(2):140-9. doi:10.1111/j.1600-0412.2010.01030.x

7. Nybo AA, Wohlfahrt J, Christens P, Olsen J, Melbye M. Is maternal age an independent risk factor for fetal loss? *West J Med*. Nov 2000;173(5):331. doi:10.1136/ewjm.173.5.331

8. American College of O, Gynecologists' Committee on Practice B-G. ACOG Practice Bulletin No. 200: Early Pregnancy Loss. *Obstet Gynecol*. Nov 2018;132(5):e197-e207. doi:10.1097/AOG.0000000000002899

9. Kim Y, Ganduglia-Cazaban C, Chan W, Lee M, Goodman DC. Trends in neonatal intensive care unit admissions by race/ethnicity in the United States, 2008-2018. *Sci Rep*. Dec 10 2021;11(1):23795. doi:10.1038/s41598-021-03183-1

10. Feldkamp ML, Carey JC, Byrne JLB, Krikov S, Botto LD. Etiology and clinical presentation of birth defects: population based study. *BMJ*. May 30 2017;357:j2249. doi:10.1136/bmj.j2249

11. Guglielminotti J, Landau R, Wong CA, Li G. Criticality of Maternal Complications During Childbirths. *J Patient Saf*. Dec 2020;16(4):e273-e277. doi:10.1097/PTS.0000000000000511

12. Taufer Cederlof E, Lundgren M, Lindahl B, Christersson C. Pregnancy Complications and Risk of Cardiovascular Disease Later in Life: A Nationwide Cohort Study. *J Am Heart Assoc*. Jan 18 2022;11(2):e023079. doi:10.1161/JAHA.121.023079

13. Xiao Q, Lauschke VM. The prevalence, genetic complexity and population-specific founder effects of human autosomal recessive disorders. *NPJ Genom Med*. Jun 2 2021;6(1):41. doi:10.1038/s41525-021-00203-x
