# Supplementary material for: Pregnancy complications and loss: an observational survey comparing anesthesiologists and obstetrician–gynecologists
Source: J Matern Fetal Neonatal Med. Author manuscript; Available in PMC 2025 Dec 1. (PMC11234813; doi:10.1080/14767058.2024.2311072)
Supplement: PregOutAnesthesiaProvider [file NIHMS2004406-supplement-PregOutAnesthesiaProvider.docx]

**Pregnancy Outcomes of Anesthesia Providers**

Please complete the survey below. Thank you!

Please confirm that you are an anesthesia provider. Yes No

If NO, please close the window and contact the study team member in which you received the email and link from to obtain the correct survey link.

**Demographics/General Information**

What is your current age? 25-30 years old

31-35 years old

36-40 years old

41-45 years old

46-50 years old

51-55 years old

56-60 years old

>61 years old

Race American Indian or Alaska Native

Asian

Black or African American White or Caucasian

Native Hawaiian or Other Pacific Islander Other

Prefer not to answer

Ethnicity Non-Hispanic

Hispanic

Prefer not to answer

Subspecialty Cardiac

Chronic Pain Critical Care Palliative Care Pediatric Obstetric Neurosurgical

Regional and Acute Pain Medicine Transplant

Trauma Other None

Please explain.

How many years have you worked in anesthesia, 0-5 years

INCLUDING residency? 6-10 years

11-15 years

16-20 years

21-25 years

>25 years

How would you describe your anesthesiology practice? Academic

Private Practice Per Diem

Other

Please explain.

What percentage of your cases do you supervise 0-25%

residents/CRNAs/AAs? 26-50%

51-75%

76-100%

I do not supervise

What percentage of your attending anesthesiologist 0-25%

colleagues are female? 26-50%

51-75%

76-100%

Do you recall learning about potential risks to Yes

pregnancy related to your specialty? No

If yes, in what context? (Select all that apply) Formal grand rounds lecture

Institutional training (i.e. learning module) Informal conversation

Your own research

Do not recall exact experience

Are you aware of a policy at your institution for Yes

support for pregnant employees? No

Are you aware of a policy at your institution for Yes

education on specialty related risks for pregnant No employees?

**Pregnancy Specific Questions**

Are you currently using or have you ever used Yes

reproductive assistance? No

If yes, please check all that apply. Oral medication(s) In vitro fertilization

Intrauterine insemination Surrogacy

Other

Please explain.

How many rounds of oral medication for reproductive assistance have you completed?

Oral medication: Yes

Did this result in a pregnancy? No

How many rounds of in vitro fertilization have you completed?

In vitro fertilization: Yes

Did this result in a pregnancy? No

How many rounds of intrauterine insemination have you completed?

Intrauterine insemination: Yes

Did this result in a pregnancy? No

How many attempts at surrogacy did you complete?

Surrogacy: Yes

Did this result in a pregnancy? No

Have you ever been pregnant? Yes

No

Gravidity?

Parity?

How many pregnancies have had associated complications (including losses)?

| How old were you at the time of your first pregnancy? | 15-20 |
| --- | --- |
|  | 21-25 |
|  | 26-30 |
|  | 31-35 |
|  | 36-40 |
|  | 41-45 |
|  | >45 |

At the time of your first pregnancy, what level of Pre-residency

training were you at? Residency

Fellowship Attending

Other (i.e. gap year)

When pregnant, have you ever experienced any of the Subchorionic hematoma following complications? (Please check all that apply) Placental abruption

Preeclampsia/eclampsia Preterm labor

Preterm rupture of membranes Uterine rupture

Other None

Please explain.

**Please answer the following questions for each complication you have checked above.**

Subchorionic hematoma: Yes

Did you experience the complication while at work? No

Subchorionic hematoma: Yes

Did you alert your employer of the complication? No

Subchorionic hematoma: Male

Was your supervisor at the time male or female? Female

Subchorionic hematoma: Yes

Were you able to leave work and seek medical care? No

Subchorionic hematoma: Yes

Did you request a leave of absence for this No complication?

Subchorionic hematoma: Yes

Were you granted the leave of absence? No

Placental abruption: Yes

Did you experience the complication while at work? No

Placental abruption: Yes

Did you alert your employer of the complication? No

Placental abruption: Male

Was your supervisor at the time male or female? Female

Placental abruption: Yes

Were you able to leave work and seek medical care? No

Placental abruption: Yes

Did you request a leave of absence for this No complication?

Placental abruption: Yes

Were you granted the leave of absence? No

Preeclampsia/eclampsia: Yes

Did you experience the complication while at work? No

Preeclampsia/eclampsia: Yes

Did you alert your employer of the complication? No

Preeclampsia/eclampsia: Male

Was your supervisor at the time male or female? Female

Preeclampsia/eclampsia: Yes

Were you able to leave work and seek medical care? No

Preeclampsia/eclampsia: Yes

Did you request a leave of absence for this No complication?

Preeclampsia/eclampsia: Yes

Were you granted the leave of absence? No

Preterm labor: Yes

Did you experience the complication while at work? No

Preterm labor: Yes

Did you alert your employer of the complication? No

Preterm labor: Male

Was your supervisor at the time male or female? Female

Preterm labor: Yes

Were you able to leave work and seek medical care? No

Preterm labor: Yes

Did you request a leave of absence for this No complication?

Preterm labor: Yes

Were you granted the leave of absence? No

Preterm rupture of membranes: Yes

Did you experience the complication while at work? No

Preterm rupture of membranes: Yes

Did you alert your employer of the complication? No

Preterm rupture of membranes: Male

Was your supervisor at the time male or female? Female

Preterm rupture of membranes: Yes

Were you able to leave work and seek medical care? No

Preterm rupture of membranes: Yes

Did you request a leave of absence for this No complication?

Preterm rupture of membranes: Yes

Were you granted the leave of absence? No

Uterine rupture: Yes

Did you experience the complication while at work? No

Uterine rupture: Yes

Did you alert your employer of the complication? No

Uterine rupture: Male

Was your supervisor at the time male or female? Female

Uterine rupture: Yes

Were you able to leave work and seek medical care? No

Uterine rupture: Yes

Did you request a leave of absence for this No complication?

Uterine rupture: Yes

Were you granted the leave of absence? No

Other complication: Yes

Did you experience the complication while at work? No

Other complication: Yes

Did you alert your employer of the complication? No

Other complication: Male

Was your supervisor at the time male or female? Female

Other complication: Yes

Were you able to leave work and seek medical care? No

Other complication: Yes

Did you request a leave of absence for this No complication?

Other complication: Yes

Were you granted the leave of absence? No

Have you experienced pregnancy loss/intrauterine fetal Yes demise? No

**Please indicate the number of losses you have experienced in each stage of pregnancy:**

Number of losses in the first trimester:

Number of losses in the second trimester:

Number of losses in the third trimester:

**Please indicate the number of losses you have experienced in each stage training:**

Number of losses pre-residency:

Number of losses during residency:

Number of losses during fellowship training:

Number of losses as an attending:

Number of losses at another time (i.e. gap year):

**Please answer the following questions regarding any pregnancy losses you may have experienced. If you have experienced more than one loss, please answer for each one in the**

**text box provided.**

Did you alert your employer of the loss?

Was your supervisor at the time male or female?

Were you able to leave work to seek medical care?

Did you request a leave of absence following any of Yes

your pregnancy losses? No

If yes, were you granted the leave of absence? (If you have experienced more than one loss, please answer

separately for each leave of absence you requested.)

Did you have a pregnancy associated with congenital Yes

anomalies? No

**Please indicate the number of pregnancies you have had associated with each congenital**

**anomaly.**

Congenital cardiac defect

Hydrocephalus

Bronchopulmonary dysplasia

Tracheoesophageal fistula

Duodenal atresia

Imperforate anus

Gastroschisis/Oomphalocele

Club foot/other limb dysplasia

Cleft lip/palate

Did you have a pregnancy associated with a congenital Yes anomaly not listed above? No

If yes, please indicate the congenital anomaly and number of pregnancies.

Did you have a pregnancy associated with a genetic Yes

syndrome? No

**Please indicate the number of pregnancies you have had associated with each genetic**

**syndrome.**

Trisomy 21

Trisomy 15

Trisomy 18

Turner syndrome

Pierre Robin syndrome

Williams syndrome

Crouzon syndrome

Goldenhar syndrome

Did you have a pregnancy associated with a genetic Yes

syndrome not listed above? No

If yes, please indicate the genetic syndrome and number of pregnancies.

**Baby Specific Questions**

Have you had a premature baby? Yes No

**Please indicate the number of premature babies you have had at each gestational age.**

24-28 Weeks

29-32 Weeks

33-36 Weeks

Did any of your babies have a NICU stay? Yes No

How many of your babies had a NICU stay?

Were any of these babies multiple gestation? Yes No

If yes, were they: Twins

Triplets

>3 babies

**Please indicate how many babies have had the following lengths of NICU care.**

Less than 1 week

Less than one month

1-3 months

3-6 months

Longer than 6 months

Did any of your babies require respiratory support in Yes

the NICU? No

Endotracheal tube

CPAP

Nasal cannula
