# Supplementary figures and images for: Pregnancy complications and loss: an observational survey comparing anesthesiologists and obstetrician–gynecologists
Source: J Matern Fetal Neonatal Med. Author manuscript; Available in PMC 2025 Dec 1. (PMC11234813; doi:10.1080/14767058.2024.2311072)

**Supplemental Figure 1:** Gravidity and parity by provider type


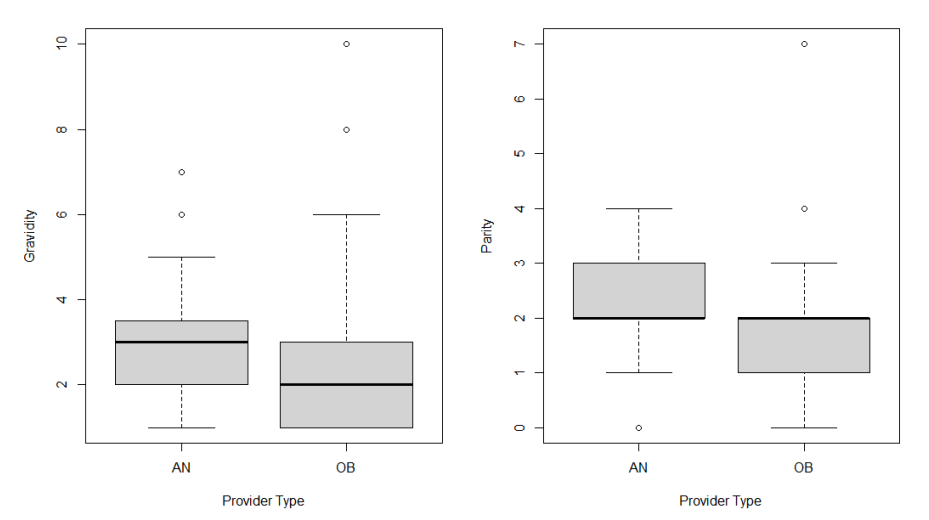

Supplement: SuppFig1 [file NIHMS2004406-supplement-SuppFig1.docx]
